# A New Method of Preserving Museum Specimens

**Published:** 1904-05-28

**Authors:** 


### A NEW METHOD OF PRESERVING MUSEUM SPECIMENS.

DR. HUGH GALT,[1] after a series of experiments made in the Glasgow Samaritan Hospital for Women, advises the use of the following solution as a medium for preserving museum specimens :—Common salt 5 ozs., potassium nitrate 1 oz , chloral hydrate 1 oz., water 100 ozs. For this several advantages are claimed. The cost is only about 4d. per gallon as compared with 2s. 6d. or more incurred by the alcohol or formalin method. The colour of the specimen is retained and is not apparently affected even by bright sunlight. The salts can be kept in the solid form and the solution prepared as desired, thus economising storage space and porterage. The preparatory treatment in this method is the same as in the old method of preservation in alcohol.

[1] Trans. Glasgow Med. Chirurg. Soc , Vol. IV.

